# The sustainability of health interventions implemented in Africa: an updated systematic review on evidence and future research perspectives

**DOI:** 10.1186/s43058-025-00716-x

**Published:** 2025-04-08

**Authors:** Ucheoma Nwaozuru, Patrick Murphy, Ashley Richard, Chisom Obiezu-Umeh, Thembekile Shato, Ifeoma Obionu, Titilola Gbajabiamila, David Oladele, Stacey Mason, Bryce P. Takenaka, Lateef Akeem Blessing, Alexis Engelhart, Susan Nkengasong, Innocent David Chinaemerem, Onyekachukwu Anikamadu, Ebenezer Adeoti, Pranali Patel, Temitope Ojo, Olufunto Olusanya, Donna Shelley, Collins Airhihenbuwa, Gbenga Ogedegbe, Oliver Ezechi, Juliet Iwelunmor

**Affiliations:** 1 Department of Implementation Science, Wake Forest University School of Medicine, Winston-Salem, NC USA; 2 Department of Behavioral Science and Health Education, Saint Louis University, College for Public Health and Social Justice, Saint Louis, MO USA; 3 Department of Medical Social Sciences, Center for Dissemination and Implementation Science Feinberg School of Medicine, Northwestern University, Evanston, IL USA; 4 Brown School at Washington University in St. Louis, Saint Louis, MO USA; 5 Clinical Sciences Department, Nigerian Institute of Medical Research, Lagos, Nigeria; 6 Washington University in St. Louis School of Medicine, Saint Louis, MO USA; 7 Department of Social and Behavioral Sciences, Yale School of Public Health, New Haven, CT USA; 8 London School of Hygiene and Tropical Medicine, London, UK; 9 Sobani Research Consult, Port Harcourt, Nigeria; 10 Department of Epidemiology, University of Alabama at Birmingham, Birmingham, AL USA; 11 School of Global Public Health, New York University, New York, NY USA; 12 Global Research Against Non-Communicable Disease Initiative, Georgia State University, Atlanta, GA USA; 13 New York University, Langone Health, New York, NY USA; 14 Division of Infectious Diseases, School of Medicine, Washington University in St. Louis, Saint Louis, MO USA

## Abstract

**Background:**

Sustaining evidence-based interventions in resource-limited settings is critical to optimizing gains in health outcomes. In 2015, we published a review of the sustainability of health interventions in African countries, highlighting gaps in the measurement and conceptualization of sustainability in the region. This review updates and expands upon the original review to account for developments in the past decade and recommendations for promoting sustainability.

**Methods:**

First, we searched five databases (PubMed, SCOPUS, Web of Science, Global Health, and Cumulated Index to Nursing and Allied Health Literature (CINAHL)) for studies published between 2015 and 2022. We repeated the search in 2023 and 2024. The review was conducted in accordance with the Preferred Reporting Items for Systematic Review and Meta-Analysis guidelines. Studies were included if they reported on the sustainability of health interventions implemented in African countries. Study findings were summarized using descriptive statistics and narrative synthesis, and sustainability strategies were categorized based on the Expert Recommendations for Implementing Change (ERIC) strategies.

**Results:**

Thirty-four publications with 22 distinct interventions were included in the review. Twelve African countries were represented in this review, with Nigeria (*n* = 6) having the most representation of available studies examining sustainability. Compared to the 2016 review, a similar proportion of studies clearly defined sustainability (52% in the current review versus 51% in the 2015 review). Eight unique strategies to foster sustainability emerged, namely: a) multi-sectorial partnership and developing stakeholder relationships, b) tailoring strategies to enhance program fit and integration, c) active stakeholder engagement and collaboration, d) capacity building through training, e) accessing new funding, f) adaptation, g) co-creation of intervention and implementation strategies and h) providing infrastructural support. The most prevalent facilitators of sustainability were related to micro-level factors (e.g., intervention fit and community engagement). In contrast, salient barriers were related to structural-level factors (e.g., limited financial resources).

**Conclusions:**

This review highlights some progress in the published reports on the sustainability of evidence-based intervention in Africa. The review emphasizes the importance of innovation in strategies to foster funding determinants for sustainable interventions. In addition, it underscores the need for developing contextually relevant sustainability frameworks that emphasize these salient determinants of sustainability in the region.

**Supplementary Information:**

The online version contains supplementary material available at 10.1186/s43058-025-00716-x.

Contributions to the literature

- Sustainability is an important indicator of implementation success, yet it is challenged by limited strategies to ensure that interventions last. This systematic review provides an overview of strategies that work well and includes some recommendations for exploring innovative sustainability strategies.
- This study contributes to understanding how and why interventions implemented in African countries are sustained.
- The review indicates the need for metrics and assessments of sustainability that leverage assets that exist in the African context while accounting for unique challenges that may impede the long-term implementation of interventions in the region.

## Background

The sustainability of interventions continues to gather momentum in implementation science as a critical translational research step essential to achieving lasting health effects [1, 2]. Defined as “the continued use of intervention components and activities for the continued achievement of desirable health outcomes within the population of interest” [3, 4], sustainability remains an important yet understudied topic [5]. As the world increasingly focuses on leaky research pipelines, with nearly 50% of studies not sustained following initial implementation [6–8], literature on how to sustain evidence-based interventions has become highly sought after [9, 10]. Sustainability is increasingly seen as a dynamic process incorporating adaptation, continuous learning, capacity building, changes, and evolutions due to complex and changing real-world settings and health systems, and not a static process or an “endgame” [2, 9, 10].

Despite the consensus on the importance of sustainability to maximize the public health impact of evidence-based interventions (EBIs) [9], evidence on the process of sustainability or the sustained use of evidence-based interventions across settings, populations, and health remains elusive [11, 12]. Available studies have elucidated some of the barriers and facilitators of sustainability [13]. However, what is especially lacking is knowledge of the processes guiding sustainability, including multiple unknown perspectives (i.e., planning for sustainability) that might turn out to be highly important [14–16]. Furthermore, published studies have highlighted the need for more evidence on a consistent measure of sustainability and how to improve the sustainability of health interventions [2, 17]. In Africa, despite the rapid growth in health innovations [18] and public health gains (e.g., increasing life expectancy and the decline in maternal and child death [19]), the region continues to lag in major health indicators compared to other regions worldwide. African countries continue to be riddled with the double burden of communicable and non-communicable diseases [20, 21] and account for a quarter of the global disease burden [22, 23]. The lag in the public health outcomes and challenges to obtaining optimal health in the region are partly attributed to a weak health system bludgeoned by low health expenditure [19, 24], low workforce [19, 24], and poor infrastructure [25]. It is important to note that there are some peculiarities across countries in the region. In light of these pervasive public health challenges, considerable resources and efforts have been dedicated to developing and implementing several public health interventions in the region, which have been proven to be efficacious [26–28]. However, similar to other regions worldwide, these EBIs are frequently not sustained [9, 29]. The poor sustainability of EBI leaves communities and organizations struggling with the issues that the EBI was intended to address, wastes investment in implementation, and can diminish community trust and buy-in for future programs [12, 30]. Beyond the impact of sustainability on public health, the limited sustainability of EBI poses an ethical dilemma for a region that is in need of more sustained public health gains.

In an attempt to understand and characterize the sustainability landscape in African countries, Iwelunmor and colleagues conducted a systematic review in 2015 that explored the sustainability of health interventions in African countries [31]. The findings highlighted a considerable need for clearer definitions for sustainability in 20 out of 41 studies included in the review [31]. The review further highlighted that community ownership and engagement were important facilitators for intervention sustainability. At the same time, limited in-country financial resources and societal upheavals were barriers that influenced the sustainability of interventions in Africa [30]. Since the review was published in 2015, it is noteworthy that there has been extensive advancement in understanding how to define and measure sustainability and typologies of sustainability strategies [9, 10, 32]. For instance, the systematic review by Lennox and colleagues focused on identifying approaches used to assess and influence sustainability in healthcare [32], and the review by Shelton and colleagues examined the conceptual and methodological issues in studying sustainability and factors that influence the sustainability of public health intervention [9]. Another review by Hailemariam and colleagues focused on identifying sustainability strategies [10]. Nonetheless, the field needs more guidance on sustaining evidence-based interventions, particularly in resource-constrained settings.

To advance the sustainability of EBIs in African countries, it is crucial to identify contextual factors that influence sustainability and to develop a conceptual framework to improve future sustainability processes and overall implementation research and practices in the region. Whether the notion of the “*fragmented and underdeveloped”* nature of sustainability in African countries [17, 31] still holds remains unknown in the region. Consequently, this systematic review builds upon the previous review published in 2015 [31] and aims to expand the knowledge on the sustainability of public health interventions implemented in Africa.

## Methods

This systematic review updates the original review conducted in 2015 [31], and the review was conducted according to the Preferred Reporting Items for Systematic Reviews and Meta-Analyses (PRISMA) guidelines [33] see Supplementary File 1. The protocol is registered with PROSPERO (registration number CRD42021243456).

### Eligibility criteria

Adapted from the 2015 systematic review [31], the inclusion criteria were: i) peer-reviewed studies focused on health interventions; ii) studies that reported on sustainability, defined using the five characteristics based on Moore et al. [34, 35]; and iii) evidence-based interventions implemented in any African country.

Informed by the criteria of the original systematic review [31] and other reviews on sustainability [10, 36], the exclusion criteria were as follows: i) studies that did not examine sustainability using any quantitative or qualitative research methodologies; ii) studies focusing only on the initial implementation phase without assessing sustainability; iii) non-empirical evidence; iv) studies with insufficient information to determine whether inclusion or exclusion criteria were met); v) generic reports that did not focus on a specific evidence-based intervention; and vi) review papers, conference abstracts, dissertations, and non-empirical publications such as commentaries, case studies, letters, posters, and conference reports.

### Search strategy

We searched five databases: PubMed, SCOPUS, Web of Science, Global Health, and Cumulated Index to Nursing and Allied Health Literature (CINAHL), from the last date of publications reviewed in the 2015 systematic review [31] until May 10th, 2022, and repeated on July 9th, 2023 and again on January 10th, 2024.

In consultation with a medical librarian and with guidance from the 2015 systematic review conducted by the research team, one of the authors (UN) developed different search strategies for each database to harmonize the unique indexing terms and functions across the databases. The expanded search strategy used terms related to sustainability, health interventions, and Africa. The search was limited to publications written in English, and we did not include grey literature. In addition, a keyword search of Google Scholar and the review of bibliographies of all selected articles and relevant reviews were performed to ensure literature search saturation.

### Study screening and selection

All studies identified were exported to EndNote software with duplicate removal on import. Three authors (PM, UN, AR) independently screened the retrieved article titles and abstracts according to the inclusion and exclusion criteria. Articles deemed eligible following title and abstract were included for the full-text review using the inclusion and exclusion criteria. One of the reviewer (UN) resolved disagreements through discussions with the other reviewers until a consensus was reached.

### Data extraction

Three authors (PM, UN, AR) independently extracted data using a piloted data extraction form (See Supplementary File 2). The extracted data included descriptive information about the article, including the first author's name and year of publication, country of study, study setting, study design and methods, participants’ characteristics (age, sample size), guiding theory/framework, intervention description, study timeline, the definition of sustainability, the unit of analysis, and study findings (barriers and facilitators to sustainability, and determinants of sustainability). Discrepancies and ambiguities with data extraction were resolved through discussion and consultation with another member of the review team (CO).

### Synthesis

The synthesis of the data extracted from the publications occurred using descriptive summaries and inductive narrative analysis. Descriptive statistics (frequencies and percentages) were used to summarize key study characteristics. In addition, we descriptively summarized key study characteristics, such as area of study, reporting of implementation outcomes, definition, and measures of sustainability.

We used an inductive narrative synthesis approach to summarize textual data extracted from the study. This narrative synthesis comprised: (i) developing a preliminary synthesis using tabulation, translating data through thematic analysis of data, and vote counting of emergent themes; (ii) exploring relationships within and between studies; and (iii) assessing the robustness of the synthesis [37]. Through the narrative synthesis process, we identified recurrent themes, and articles were categorized based on similarities and differences in settings, participants, public health outcomes, and study findings. In addition, we identified the most relevant barriers and facilitators to sustainability in African countries through inductive thematic analysis to reflect emerging themes from the manuscripts. These features were grouped into themes that captured patterns of barriers, facilitators, and determinants of sustainability in Africa. Also, sustainability strategies used in the studies were identified guided by the Expert Recommendations for Implementing Change (ERIC) strategies [38, 39] and the modified ERIC for sustainment [40].

### Quality assessment

Two authors (UN, CO) appraised the quality of all retained studies independently using Hawker’s Quality Assessment Checklist [41]. Details are provided in Supplementary File 3. No study was excluded even after quality appraisal, irrespective of its methodological quality, to increase the comprehensiveness of the systematic review by allowing the consolidation of all available evidence.

## Results

### Study selection

**Fig. 1:**
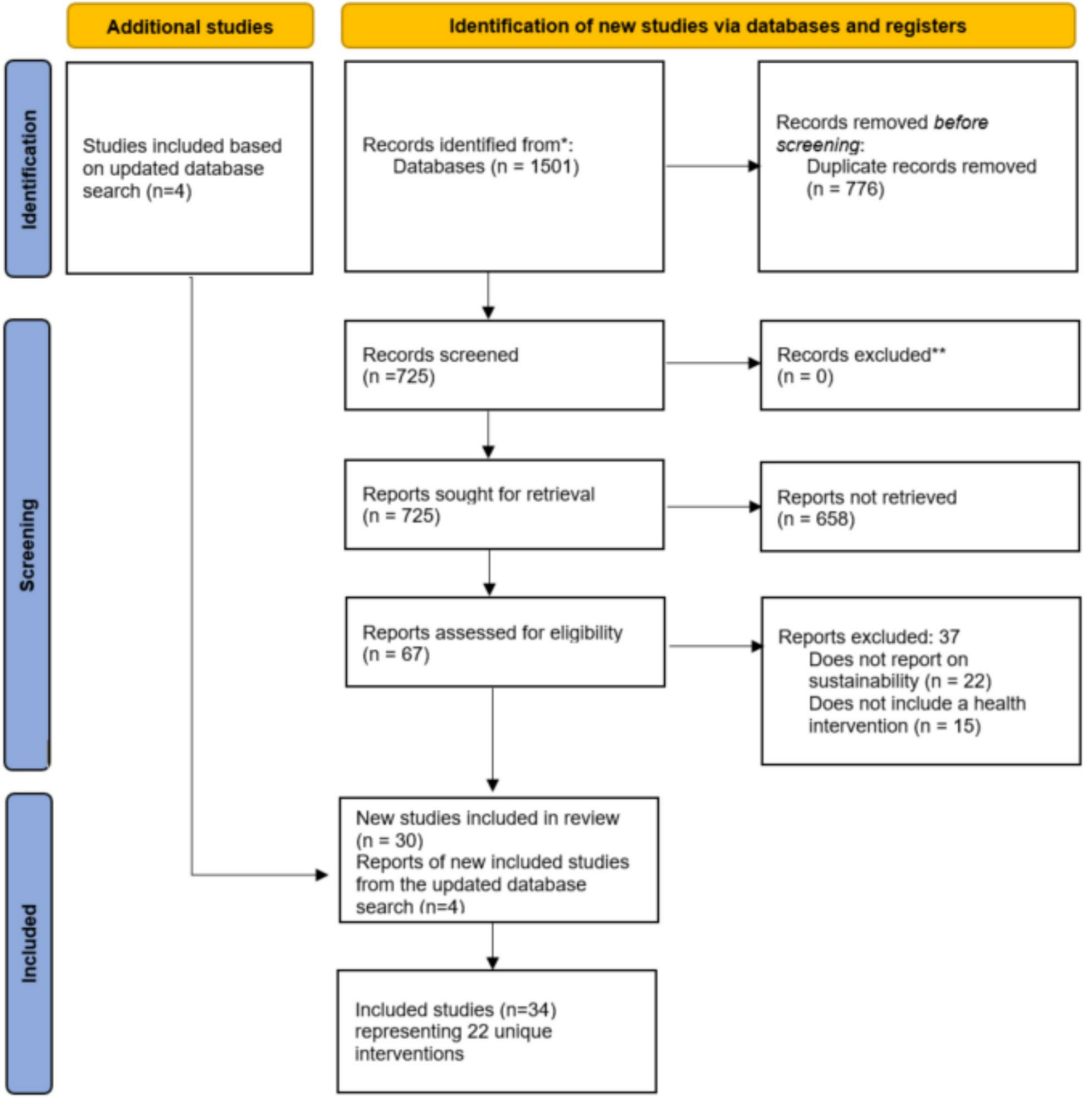
Flow chart of studies included in the review

**Fig. 2:**
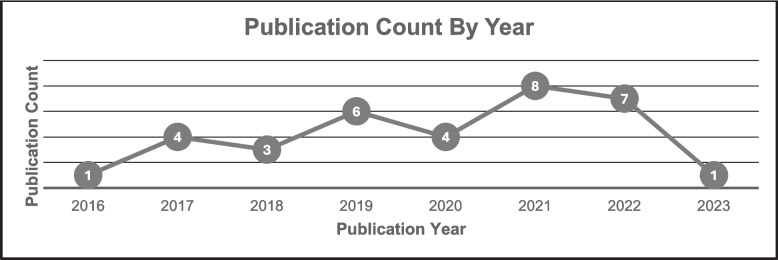
Report on the frequency of publications by year. Note: This is based on articles included in the study (*N* = 34). Some interventions were represented by more than one study

The database search yielded 1501 publications. Of these records, 776 were excluded for being duplicates. The titles and abstracts of the remaining 725 articles were screened for potential inclusion. After that, 658 were excluded, and the full text of 67 articles were reviewed. We finally selected 30 studies that met our inclusion criteria; 37 were excluded. Four additional studies were identified from the updated database search, resulting in 34 studies representing 22 unique sustainability interventions included in this review. Figure 1 shows the selection process. Included studies were published between 2016 and 2023, with the highest in 2021 (*n* = 8) and the lowest in 2016 and 2023 (*n* = 1). See Fig. 2.

### Quality assessment

Quality assessment of the studies using Hawker’s Quality Assessment Checklist. All the included studies were classified as medium or high quality, with quality scores ranging from 27 to 32. Only two studies were ranked medium [42, 43], and the remaining were ranked high [39, 44–63]. The medium-rated studies provided limited details on sampling, analysis, and recruitment strategies.

### Study characteristics

Table 1 provides details on the description of the interventions included in the review.

**Table 1:** Description of the interventions included in the review

Reference; Context	Intervention Description	Study setting	Theory or Framework used to conceptualize sustainability	Timeline of the project [Sustainability assessment Period]	Analytical Approach	Report on whether the intervention was sustained
Abamecha et al., 2021 [44]; Ethiopia	Social and behavior change communication interventions for malaria prevention. Focused on promoting malaria-preventive practices at the school and community-level	School-based	Not reported	2017–2019 [Assessment period: At the end of the program, 10–30, 2020]	Mixed-methods:Quantitative data (survey) and qualitative data (interviews) collected from officials from education and health offices, community health extension workers (HEWs), school directors, school malaria focal teachers and the program’s field officers	Not reported
Ashaba et al., 2022 [47]; Hobbs et al., 2022; Uganda	Healthy Child Uganda- MamaToto Program A maternal and child health intervention that included health system strengthening, health facility capacity strengthening, and deployment of Community Health workers (CHWs)	Multi-level intervention [district, community, and health facility]	Not reported	Study Period: 2012–2014 [Assessment Period: July–August 2018]	Mixed-methods: Sustainability-focused qualitative evaluation. Focus group discussions and in-depth interviews among CHWs, health providers (health assistants, clinicians), community development officers, and community leaders Retrospective operational review of an existing population-based database comprising all CHWs in two districts	Yes, at the time of publication Noted as retention of over 80% of volunteer CHWS
Blackstone et al., 2017 [52]; Ghana	Task-shifting strategy for hypertension (TASSH) control in Ghana	Health-facility based	Not reported	2014–2016 [Assessment period: 2017]	Mixed-methods: Concept mapping with nurses to understand community health nurses' perceptions of barriers and enablers to sustaining a task-shifting program	Unclear status in Ghana, but has been adapted for implementation in Nigeria
Busza et al., 2018 [63]; Busza et al. 2018b [64]; Zimbabwe	Zimbabwe Study for Enhancing Testing and Improving Treatment of HIV in Children. A community-based intervention offering community health worker home visits to caregivers of children living with HIV in seven high-density communities in Harare, Zimbabwe	Community-based [delivered at homes]	Not reported	[Assessment period: end of trial]	Qualitative method: Longitudinal semi-structured qualitative interviews with the 19 CHWs who delivered the ZENITH intervention at three time periods during the trial: baseline (following recruitment and training but before home visits work started), midline (after one year of implementation of the intervention), and at the end of the intervention 2 years later	No
Chelagat et al., 2021 [65]; Chelagat et al., 2019 [62]; Kenya	The Leading High-performing Healthcare Organisations’ (LeHHO). The leadership program aims to enable senior national and county management teams to address the most critical health system challenges in a devolved system of government	Multi-level Health-facility-based and community-based	Comprehensive conceptual sustainability from Iwelunmor et al. 2015 [31]	2011–2016 [Assessment period: post-training]	Qualitative method: Interviews nested within a quasi-experimental study	Partly 85% of the site sustained the intervention
Chiliza et al., 2021 [53]; South Africa	PEPFAR program to enhance linkage to care for HIV	Health-facility based	Not reported^a^ However, the background references literature on sustainability	2007–2012 [ Assessment period: October 28, 2018 to April 3, 2019]	Mixed-methods: Health facilities records and interviews conducted with health facility managers, clinical nurse practitioners, government officials, and NGO program managers	Partly
Chowdhary et al., 2022 [45]; Ethiopia	CARE TESFA program (TESFA means “hope” in Amharic). Delivered reproductive health and financial savings curricula to married girls [10–19 years] via reflective dialogues in peer-based solidarity groups	Community-based	Not reported^a^ However, the background references literature on sustainability	2010–2013 [Assessment Period: 2017- 4 years after TESFA implementation ended]	Qualitative method: Focus group discussions and in-depth interviews with study participants	Yes Noted as 88% of surveyed groups were found to still be active and using the sexual and reproductive health and economic empowerment curricula
Crocker et al., 2017 [54]; Ethiopia and Ghana	Community-led total sanitation program. Focused on addressing open defecation that triggers emotions to generate a collective demand for sanitation within a community	Community-based	Not reported	Ethiopia: 2012–2013 Ghana:2013–2014 [Assessment: One after completion of the intervention]	Quantitative method Ethiopia– > quasi-experimental design. Survey data was collected at baseline, immediately after the interventions (midline), and again one year later (end of trial) Ghana – > Survey data collected immediately after the interventions (midline) and again one year later (end of trial))	Not reported
Dharmayat et al., 2019 [51]; Malawi	The Supporting Low-cost Intervention For Disease Control (Supporting LIFE). The SL eCCM App was developed as an Android a smartphone application that replicates the Community Case Management program decision aid tool routinely used by Health Surveillance Assistants (HSAs) in Malawi. This app enables HSAs to enter the same information (usually gathered using a paper-based CCM form), including personal details (e.g., gender), clinical symptoms (e.g., fever) and clinical measurements (e.g., breathing rate). Data is entered directly into the application. The app then provides the user with the recommended treatment for the child, such as treatment at home with medication or referral to a higher-level clinical facility	Health-facility-based	Shediac-Rizkallah and Bone framework conceptualizing program sustainability [12]	4-year program [Assessment period: At the end of the implementation -January and March 2017]	Qualitative method: Interviews among stakeholders in Malawi, including district health officers, zonal health officer, Integrated Disease Surveillance, and Response (IDRR) programmer, Central Monitoring and Evaluation Division (CMED) officer, ministry of health, senior member involved in research and NGO members	Unclear
Fontanet et al., 2020 [55]; Scott et al., 2021; Fong et al. 2022; Zambia	Maternity Homes Access in Zambia project. Community-driven maternity waiting homes model in rural Zambia. This involved the construction of 10 maternal waiting homes(MHW) adjacent to rural health centers able to provide obstetric care for uncomplicated deliveries and within 2 hour to a referral hospital equipped to care for women experiencing obstetric complications. The 20 MWHs met the three main pillars of the Infrastructure, equipment, and supplies to ensure a safe, comfortable, and functional structure; Policies, management, and finances to ensure local oversight and sustainability of the homes; Linkages and services to ensure integration with the formal health system	Community-based	Scheirer and Dearing’s framework for the sustainability of public health programs [66]	[Assessment period: 2016–2018; 3-time points: immediately following intervention lunch, during the intervention and after implementation phase-out]	Qualitative method: Focus group discussions and in-depth interviews with community members, and community health volunteers	Not reported
Graham et al., 2022 [50]; Graham et al. 2019 [67]; Nigeria	Nigeria Oxygen Implementation project to improve hospital oxygen systems in 12 hospitals in southwest Nigeria. 12 hospitals were provided pulse oximeters and training and oxygen delivery systems The multifaceted intervention (full oxygen system) was delivered at the cluster (hospital) level and involved (1) a standardized oxygen equipment package, (2) clinical education and support, (3) technical training and support, and (4) infrastructure and systems support	Health facility-based	Not reported	2015–2021 [Assessment Period: medium-term assessment-2018–2020]	Mixed-methods: Cross-sectional facility assessments, clinical audits, WHO facility assessment tools, direct observation, recording of informal feedback from technicians, cost information, and clinical outcome data	No
Hirschhorn et al., 2021 [46]; Rwanda	Under-5 mortality reduction evidence-based intervention implemented before and during the period of focus (2000–2015), with pneumococcal vaccine (PCV) as the case study. Various evidence-based interventions focused on amenable under-5 mortality in Rwanda	Health facility-based	Hybrid Implementation Research (IR) Framework that includes aspects of EPIS, RE-AIM, and CFIR	2000–2015 [Assessment period: End of trial]	Qualitative method: Key informant interviews from implementing non-governmental organizations, multilateral organizations, and donor organizations who had been involved in partner-supported or partner-led activities	Yes Full vaccination rates with PCV remained consistently high from introduction through the end of the study period and beyond, with coverage at 97–98% since 2010
Katuramu et al., 2020 [48, 68]; Uganda	The START-ART rapid ART initiation program included training healthcare workers and introducing additional resources to support the program	Health facility-based	Capability, opportunity, or motivational components model (COM-Model) [69]	April 2013 to June 2015 -Intervention period 1 August 2015 to 31 July 2016- Sustainability period. [Assessment period: 4 October 2017 to 15 February 2018]	Mixed-methods: This included a cross-sectional patient record review and key informant interview. Key informant interviews were conducted at one facility that sustained the intervention and one that did not	Partly Engagement of some of the participants in ART adherence
Moore et al., 2023 [43]; Stark et al., 2020; Burkina-Faso	Un Oeuf intervention. The intervention aimed to increase egg consumption in children ages 6–24 months through a culturally tailored Behavioral change communication strategy to improve poultry production and empower women. Messaging encouraged women to feed enrolled children an egg every day	Community-based	Not reported	[Assessment period: data collected at the end line and immediately following the endline, all in 2019]	Mixed-methods: Combining quantitative and qualitative Data. This included cross-sectional survey and focus group discussions exploring the barriers and facilitating factors and the sustainability and scalability of the intervention	Yes Continued use of program activities
Moucheraud et al., 2017 [56]; Malawi, Zambia, and Zimbabwe	Electronic health information systems (EHIS) project	Health facility-based	Sustainability framework that maps the determinants of sustainability based on recommendations from Bossert 1990, Stirman et al. 2012 [17], Gruen et al. 2008 [11], and Scheirer 2005 [71] (*n* = 1)	The project started in 2001 in Malawi, 2009 in Zambia, and 2009 in Zimbabwe [Assessment period: 2013]	Qualitative method: Interviews with major stakeholders involved in ongoing donor-funded projects for strengthening HIV care through EHIS	Unclear
Mwale et al., 2021 [57] Mwale et al., 2021b [70]; Malawi	CARE’s Community Score Card© Malawi, as part of the Maternal Health Alliance Project (MHAP) a social accountability approach that aids in assessing, monitoring, and evaluating government services with a focus on healthcare	Community-based	Not reported However, the background references literature on sustainability	2012–2015 [Assessment period: ~ 2.5 years after the end of the formal MHAP project]	Mixed-methods: Cross-sectional design comparing the sustainability of various partner-led approaches, focus groups with members of Community Health Advisory Groups (CHAGs) and youth groups, and semi-structured interviews with local and district government officials, project staff, and national stakeholders to understand how and in what form intervention activities are continuing	Yes Community adoption/ownership of the program
Obi-Jeff et al., 2022 [58]; Nigeria	Immunization Reminder and Information SMS System (IRISS) in Kebbi State. IRISS used SMS to inform and educate the public about the importance of immunization and remind caregivers/parents of their child’s immunization schedules, including the vaccination schedules of health facilities in their locality	Community-based	RE-AIM [71]	May 20,2019-May 31, 2020 [Assessment period: June 2020]	Qualitative method: Focus Group Discussions, In-depth Interviews (IDIs), and Key Informant Interviews with community members, government program managers, government health workers, and policymakers	No
Onwujekwe et al., 2019 [59]; Nigeria	A Free Maternal and Child Health program The National Health Insurance Scheme (NHIS)-MDGs Free Maternal and Child Health Program was an intervention to address the high mortality among women and children. The intervention program provided access to pregnant and children under-5 to free health care services from primary health centers and then referred to selected general hospitals when there were complications	Health facility-based	Health system building blocks [72]	2009–2015 The program started in 2009 with six pilot states and was scaled-up to 12 states in phases 2, 3, and 4 [Assessment period: Feb-Aug 2016]	Qualitative method: In-depth interviews with NHIS, OSSAP‑MDG, HMOs, Public health facilities, state/local governmentand communities; document review, including policy documents, program implementation reports and other relevant reports	No
Prasad et al., 2022 [42]; Tanzania	The Program to Reduce Maternal Deaths in Tanzania. This task-sharing intervention focused on increasing access to maternal and child health services by utilizing assistant medical officers, facility improvement intervention to increase access to high-quality services, and demand generation activities This involved leveraging task sharing, which allowed certain cadres of associate clinicians—such as assistant medical officers (AMOs)—to provide comprehensive emergency obstetric and newborn care (CEmONC)	Health facility-based	Not reported	2006–2019 [Assessment period: During implementation and end of Implementation]	Mixed-method approach: This included the documentation of operational performance and outcomes in program-supported facilities, reproduction health surveys, health facilities assessment, clients and providers surveys, pregnancy outcome monitoring	Yes The program fully transitioned to the Government of Tanzania's oversight
Speizer et al., 2019 [73]; Speizer et al.,2019b [74]; Olumide et al., 2020 [60]; Nigeria	The Nigerian Urban Reproductive Health Initiative (NURHI). The NURHI program utilized demand generation activities [community-level outreach events, distribution of information, education, and communication (IEC) materials at public and private health facilities in the communities and through mass media, including television and radio programs] to encourage interpersonal discussion about family planning, reduce barriers, myths, social stigma, and increased approval of family planning methods	Multi-level Community-based and health facility-based	Shediac-Rizkallah and Bone [12]	Phase I implementation (2009–2014) Phase II implementation (2015-) [Assessment period: Evaluation in 2014, end of Phase I Evaluation in 2015 & 2017 of the phase II sites]	Mixed-methods: Descriptive characteristics from longitudinal data collected from the study sites, Principal component analysis to explore predictors of sustainability and in-depth interviews with service providers	No
Wickremasinghe et al.,2021 [61]; Nigeria	Village Health Worker Scheme. This involved village health workers working in their communities to promote maternal and child health. Their role involved delivering maternal, newborn, and child healthcare messages, encouraging improved health and healthcare-seeking behaviors, and undertaking basic healthcare provision, such as treating pregnant women for anemia and referring them to health facilities, promoting healthcare uptake	Community-based	Based on the literature on sustainability [Specifically, a conceptual framework informed by *Hirschhorn *et al*. 2013, Larson *et al*. 2014*, *Torpey *et al*. 2010*, and *WHO and ExpandNet (2010)*	2016−2019 [Assement period: 2017 and 2018. Multiple assessments: at setting-up phase (Sept 2017]; consolidation phase (Jan-Feb 2018); and mature phase (Nov-Dec-2018)]	Mixed-methods: Descriptive characteristics for the longitudinal data from 2015 and 2017 from two cities in the study. Principal component analysis to explore predictors of sustainability and in-depth interviews with service provider	No
Zakumumpa et al., 2016 [75, 76]; Zakumumpa et al., 2017; Zakumumpa et al., 2018 [49]; Uganda	Uganda national ART scale-up program at public and private health facilities. Focused on promoting uptake of ART	Health-facility based	Shediac-Rizkallah and Bone Sustainability Framework [12]	2004–2009 [Assessment period:2014–2015]	Mixed-methods: Surveys among ART clinic managers, in-depth interviews with patients and clinic managers, health facilities evaluations, on-site checklists, and document review	Unclear

^a^deduced from the study introduction

#### Area of study

The review covers 22 interventions across 12 countries in Africa, representing Eastern Africa 8 (40%) [42, 44–49, 65], Western Africa 7 (35%), [43, 50, 52, 58, 59, 61, 73], and Southern Africa 5 (25%) [51, 53, 55, 57, 63]. Two multi-country interventions were excluded from the regional count but included in the total, resulting in 12 countries, Nigeria (6), Uganda (4), and Malawi (4) being the most represented.

#### Study settings

Interventions were implemented in diverse settings: 50% (*n* = *22)* interventions in health-facilities [42, 46, 48, 49, 51–53, 56, 59, 61, 67], 32% (*n* = *6) *[43, 45, 54, 55, 57, 58] interventions in community settings, one intervention each in a school (4%) [44], and one at participants' homes (4%) [63]. Three interventions were implemented in both community and health facility settings [47, 65, 74].

#### Health outcomes reported

Similar to the 2015 review, the health outcomes reported remain diverse [31]. Of the 22 interventions included in the review, 32% (*n* = *7)* focused on communicable diseases, primarily HIV [44, 48, 49, 53, 54, 56, 63]. Maternal and child health-related outcomes represented 28% (*n* = *6*) of the interventions [42, 43, 47, 55, 59, 61], followed by under-five mortality (*n* = *4*) [46, 50, 51, 58], reproductive health among women 9% (*n* = *2), *[45, 74]*,* and adolescent sexual and reproductive health 5% (*n* = *1)* [57]*.* One intervention focused on non-communicable disease control-hypertension control 5% (*n* = *1) *[52]*,* and health system improvement 5% (*n* = *1) *[65].

#### Theory or framework used

The sustainability of eleven of the included interventions (50%, 11/22) [46, 48, 49, 51, 55, 56, 58, 59, 61, 65, 74] were explicitly assessed using some form of guiding framework or theory. A variety of sustainability and implementation science models/theories and other frameworks were used. The most common framework utilized was the *Shediac-Rizkallah and Bone sustainability framework *[12], used in three studies. Other frameworks used in the sustainability assessment were *the comprehensive conceptual sustainability from Iwelunmor *et al*. *[31] (*n* = *1*), *capability, opportunity or motivational components model (COM-Model) *[69] (*n* = 1), *Health system building blocks* [72] (*n* = 1), the *Reach, Effectiveness, Adoption, Implementation, and Maintenance Framework (RE-AIM)* (*n* = *1*) [71], and *Scheirer and Dearing’s framework for the sustainability of public health programs* (*n* = 1) [66]. Three studies [46, 56, 61] developed a sustainability framework based on a combination of multiple empirical evidence or existing frameworks.

#### Types of methods used

The most common method for assessing sustainability was mixed methods (*n* = 11), followed by qualitative (*n* = 10) and quantitative (*n* = 1). Mixed methods included concept-mapping and combining surveys, database reviews, audits, interviews, and focus groups. Interviews were the primary data collection method for qualitative studies.

### Sustainability-related results

#### Timeframe of sustainability assessment

The majority of the studies, 86% (*n* = *19*) [42–45, 47–49, 51–55, 57–59, 61, 63, 67, 73] provided an exact timeframe between the implementation period and sustainability assessment, while the remaining 14% (*n* = *3)* [46, 56, 65] did not explicitly provide a timeline for the sustainability evaluation in relation to the intervention implementation. For two of these studies [46, 65] with an unclear timeframe for sustainability evaluation, it can be inferred from the study discussion that the evaluation occurred at the end of the implementation period. The other study [56] provided a date for assessment but no details on the implementation period. This study is ongoing, suggesting a potential medium-term evaluation of sustainability.

Among the 19 studies with reported timing, 12 conducted sustainability assessments at a single time point [42–45, 47, 49, 51–55, 57–59, 61, 63, 67, 68, 73], ranging from 1 month to 6 years post-implementation. The median timeframe was 1.75 years post-implementation. Seven studies [42, 43, 48, 55, 61, 63, 73] evaluated sustainability at multiple time points, typically at baseline, mid-implementation, and post-implementation.

#### Sustainability strategies

**Fig. 3:**
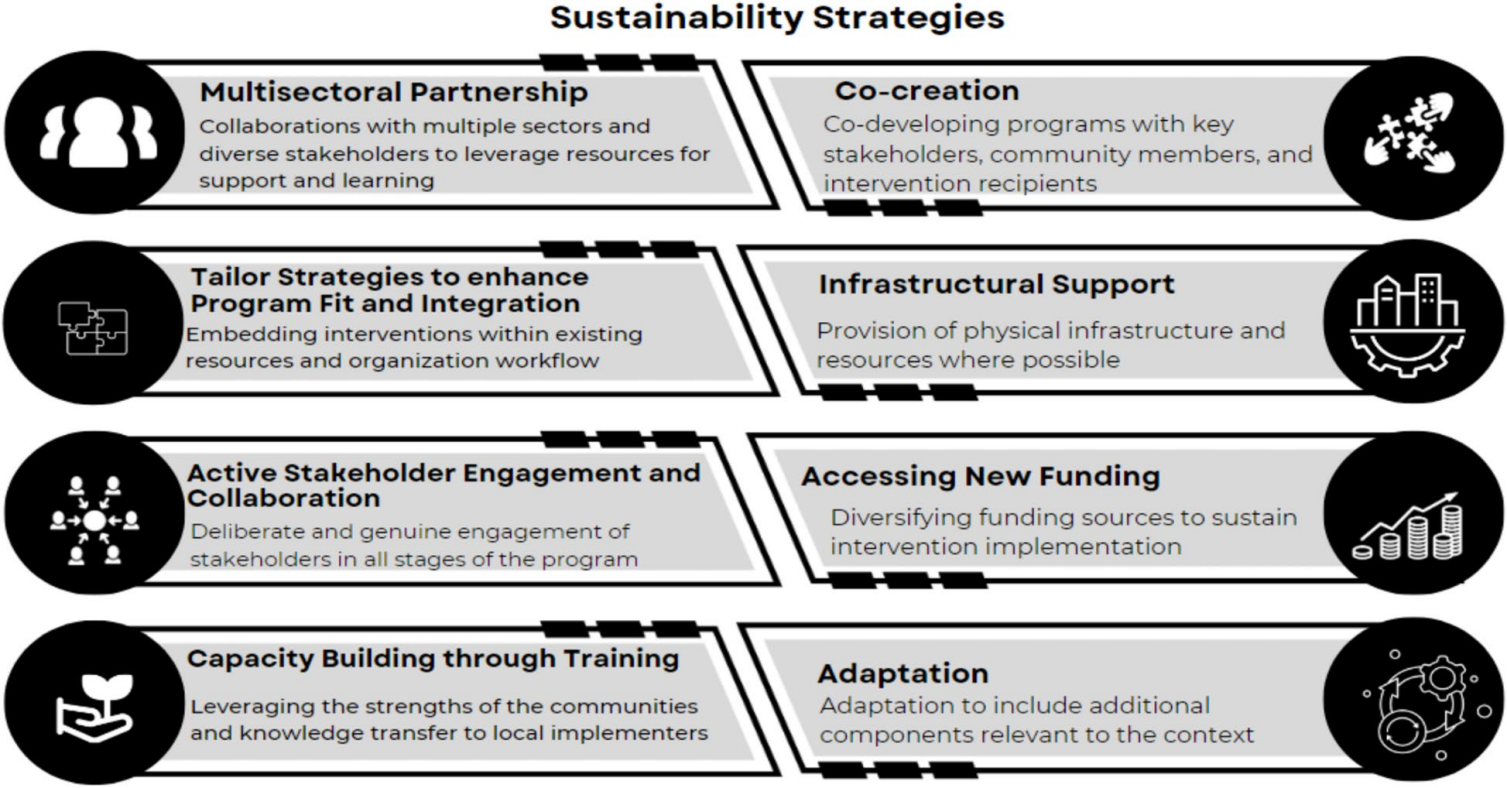
A pictorial representation of the sustainability strategies identified in the review

Twelve of the 22 included interventions (55%) explicitly stated the sustainability strategies they employed to sustain the intervention activities or health impact. Across these twelve studies, eight unique sustainability strategies were utilized. Six of these strategies align with the existing ERIC strategies [38, 39] and the modified ERIC for sustainment [40]. These included i) multi-sectorial partnership and developing stakeholder relationships, ii) tailoring strategies to enhance program fit and integration, iii) active stakeholder engagement and collaboration, iv) capacity building through training, v) accessing new funding, and vi) adaptation. Two additional themes not captured by ERIC or the modified ERIC emerged: i) co-creation of intervention and implementation strategies and ii) infrastructural support. These strategies are shown in Fig. 3.

#### Sustainability definition and outcomes reported

##### Definitions

While all the studies in this review focused on some aspects of sustainability, only 55% (*n* = *12*) of the interventions clearly defined sustainability [44, 46–48, 51, 53, 55, 56, 61, 65, 73, 75]. Among the studies with clear descriptions of sustainability, seven studies [44, 47, 49, 51, 55, 56, 65] based their definitions on previous literature. Specifically, definitions from Shediac-Rizkallah and Bone [12], Lapelle et al. [77], Moore et al. [1], Stirman et al. [17], and the World Health Organization were cited. The definition by Shediac-Rizkallah and Bone [12, 65] was the most frequently cited work, with three studies [47] using their definition verbatim. In addition, the definition by Stirman et al. [17] was cited by two studies [49, 55]. The remaining five studies [46, 48, 53, 61, 73] described sustainability based on a combination of established definitions or developed their own definitions for sustainability. Collectively, all the studies assessed sustainability, and various terms were used to describe it. This includes terms like *“sustainability”*, *“sustainment”, “maintenance”, “institutionalization”, “longevity”,* and *“continuation.”*

##### Sustainability outcomes

Most studies 55% (*n* = *12*) [44, 47, 49, 51, 52, 54, 58, 59, 61, 63, 65, 74] reported sustainability outcomes related to the continuation of program activities or components of the interventions beyond the study implementation period or funding period. Other outcomes included maintenance or improvement of health benefits to intended recipients 3% (*n* = *6*) [42, 46, 48, 53, 56, 74], fostering community ownership 18% (*n* = *4*) [42, 55, 57, 61], maintenance and upkeep of equipment 5% (*n* = *1) *[50], and 5% (*n* = *1) *scale-up of the intervention activities through replication and dissemination [45].

Despite all studies assessing sustainability outcomes, only eight (36%) [42, 43, 45, 47, 48, 53, 57, 65] explicitly stated the sustainment activities in full or in part beyond the implementation or funding period. In four interventions, sustainment was reported as the continuation of intervention activities or components. Examples of EBI activities or components of interventions sustained include retention and continued engagement of 80% of volunteer community health workers [47], continued use of and scale-up of intervention beyond the study area [45], keeping poultry farms functional to promote child nutrition [43], and continued leadership training in 85% of health facilities to promote healthcare delivery [65].

Other sustainability indicators included long-term health benefits and intervention integration. For instance, one study reported varying levels of patient retention in HIV care facilities post-PEPFAR program [53]. Additionally, the maternal and child mortality program in Tanzania was fully transitioned to the government [42], and village health workers in Nigeria were incorporated into a broader community health program [61]. In Malawi, community ownership led to the continued use of the community scorecard from the Maternal Health Alliance Project [57].

### Thematic synthesis of facilitators and barriers to sustainability

**Fig. 4:**
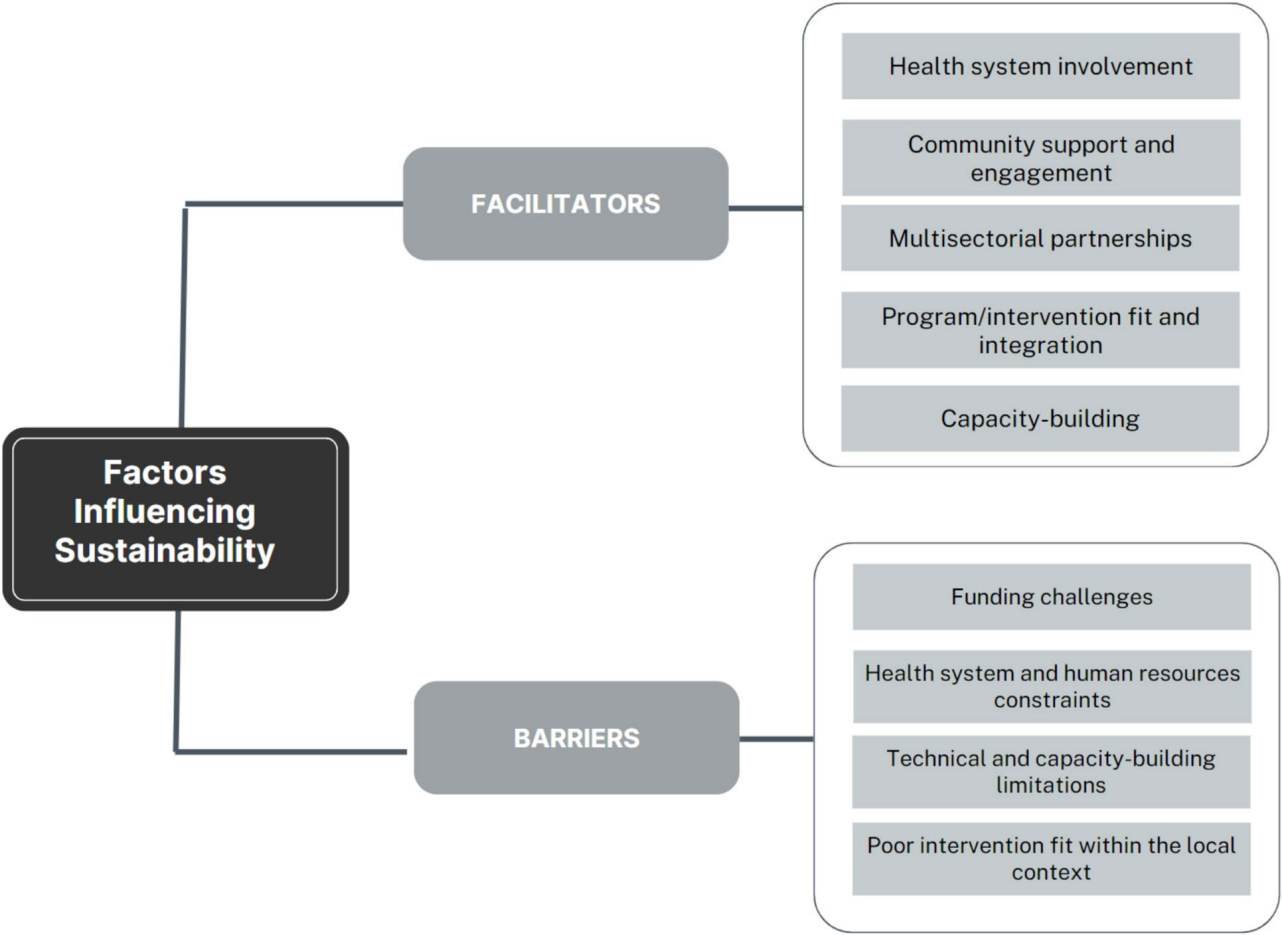
Facilitators for and barriers to sustainability

Figure 4 shows the thematic categories of barriers and facilitators identified across the studies included in the review.

#### Facilitators of sustainability

The studies identified several key facilitators for the sustainability of health interventions, which can be grouped into five main themes:

- *Health system involvement:* The active involvement and commitment of the health system and healthcare providers were identified as crucial factors for ensuring the sustainability of health interventions in six studies [46, 47, 53, 54, 57, 65]. The support of the leadership within the health system was important in fostering interventions' institutionalization [65]. Notably, broader health system leadership's financial and political commitment was highlighted as critical in achieving sustainability.
- *Community support and engagement:* Sixteen studies explicitly reported that active community engagement enabled the adoption and sustaining of the intervention components and fostered a sense of ownership among the end-users and/or recipient communities [42, 44, 46, 47, 49–51, 53–55, 58, 59, 61, 63, 65, 70]. These studies highlighted the importance of active community engagement in the planning and implementation of intervention/program activities [46, 47, 51, 54, 55, 65]. For example, one of the studies highlighted that people and relationships were crucial for intervention implementation success [53]. These factors were nurtured through community engagement, long-standing partnerships, presence, and honing interventions to leverage the values and needs of the community [53]. In addition, Fontanet et al. [55] emphasized the centrality of community members in implementing a maternal health intervention. They noted that the community's active participation, including financial contributions and involvement in building maternal waiting homes, increased community ownership and communal responsibility. This level of engagement contributed to the intervention's success and long-term sustainability.
- *Multisectoral partnerships:* Five interventions emphasized the importance of coordinated actions across multiple sectors (i.e., government, private, non-profit, and community) to address public health issues effectively and sustain intervention activities [42, 49, 51, 54, 65]. Public health interventions are inherently multi-sectorial and do not occur in vacuums. Notably, to enhance funding for implementation, some studies suggested investments from grants, health systems, and private and not-for-profit sectors. In addition, the Multisectoral partnership provides learning opportunities and enhances strategies for EBI implementation and sustainability. For instance, in an intervention in Malawi focused on addressing under-5 mortality, Dharmayat et al. [51] highlighted that the involvement of the Ministry of Health and interventional organizations provided an opportunity to leverage the strengths and expertise of these entities. In the long-term, this synergistic partnership was essential in integrating the intervention into the health system and for sustainment.
- *Program/Intervention fit and integration:* Eleven interventions highlighted the significance of intervention alignment with local resources, policies, culture, and strategic plans for sustainability or in planning for the sustainability of their interventions [44, 45, 49–52, 54, 56, 61, 64, 74]. Embedding interventions into existing healthcare systems or community programs or priorities allowed for the continuation of the intervention even when the specific project funding ended. Intervention fit involved using equipment that are efficient within the local context and can be easily repaired by individuals in the setting [50], using contextually appropriate intervention curriculum or activities [45, 54], ensuring that interventions fit within existing structures and routines in the context (i.e., clinics, education system, community settings, etc.), [44, 49, 51, 56, 61] and adaptability to enhance its alignment with the local context [56, 61].
- *Capacity building:* Nine studies identified local implementers' capacity building and training as essential facilitators of sustainability [47–52, 54, 56, 74]. These studies stressed the value of providing stakeholders and local implementers with the necessary skills, knowledge, and resources to implement the EBIs effectively. This involved equipping key stakeholders and local implementers to implement the interventions and train other individuals, creating a cascading effect of knowledge dissemination and skill development. Building local capacity fosters a sense of ownership and fosters continuity of intervention.

#### Barriers to sustainability

The studies also outlined several barriers to sustainability, categorized into four main themes:

- *Funding challenges: *Thirteen studies identified limited funding and resource constraints as major barriers to the sustainability of health interventions [44, 47, 51, 52, 54–57, 59, 61, 65, 67, 68, 74, 76]. Insufficient funds and essential resources, such as equipment, screening materials, and medications, posed significant challenges in implementing and maintaining interventions effectively [47–49, 52, 55, 57, 74]. Particularly, interventions heavily reliant on external funding, such as grants, and not integrated within existing resources faced difficulties sustaining their continuity once the external funding ended. This often resulted in the inability to retain project staff, purchase project materials, which affected the program's overall functioning [49, 52]. Additionally, inadequate financial support from government and health authorities further disrupted the continuity of health programs.
- *Health system and human resources constraints:* Six studies identified health system-level barriers, which included material and human resource constraints [52, 61, 63, 65, 68, 76]. Inadequate availability of supplies and medications in some of the health facilities where interventions were implemented were identified as challenging to the program's long-term sustainability. Some health facilities were already overstretched, partly attributed to limited-service equipment and staff shortages. For instance, Katuramu et al. [48], 68] noted stockouts of materials required for rapid CD4 testing, which negatively impacted the sustainability of an ART management program. In addition, six studies reported the shortage of health professionals at health facilities, constraining intervention implementation and long-term sustainability [52, 61, 63, 65, 68, 76]. In some of the interventions, the implementation of the intervention was a burden to the already overworked staff and health providers, who received little to no additional remuneration for the extra tasks [76]. To mitigate the challenge of understaffing in some health facilities, some interventions hired additional staff during the funded period to support implementation. However, retaining these staff members became problematic once the funding period concluded. Consequently, this led to a loss of trained personnel and institutional knowledge, and the affected staff also experienced a loss of income post-intervention period. This challenge was further exacerbated by the COVID-19 pandemic, which disrupted the health system entirely.
- *Technical and capacity-building limitations:* The complexity of some technologies and lack of proper training and support posed barriers to sustainability. This barrier was documented in four studies [51, 64, 68, 73]. While technology may offer innovative solutions, in some studies, the lack of experience and familiarity with the tools hampered sustainability over time [51]. In some cases, due to poor capacity-building, the internal implementers and/or end-users could not address technical issues with technology or interventions at the end of the funding period or after the external implementers leave [56]. In addition, inadequate training and poor technical support left the staff with a limited understanding of the program, which hindered their ability to coordinate or implement the program beyond the involvement of the external implementers [44].
- *Poor intervention fit within the local context*: The lack of proper intervention fit within the local context poses a significant challenge to their effectiveness and long-term sustainability. It was highlighted in seven studies as a salient barrier to sustainability [44, 45, 51, 52, 56, 59, 65]. When EBIs do not align with the priorities, cultural norms, or existing resources and infrastructure of the target community or organization, they are at risk of not being sustained. For instance, interventions that demand extensive resources and infrastructure may not be feasible to continue beyond the study or funding period. In addition, inadequate engagement of all communities of interest, including end-users, organizational leaders, government leaders, etc., in intervention planning and implementation particularly contributed to poor intervention misalignment, ultimately limiting sustainability [44, 65].

## Discussion

### Some advancements in the assessment of sustainability, but gaps in knowledge from prior reviews persist

We conducted a systematic review of the sustainability of public health interventions in African countries to update an earlier review published in 2015 by Iwelunmor et al. [31]. That review reported 41 studies on sustainability covering a span of 19 years from 1996 to 2015. This review sought to expand knowledge on the state of sustainability research in the African region, the progress made, and recommendations for future research explorations. This updated review includes 22 unique interventions published from 2015 to 2023, indicating continuing interest in documenting the sustainability of EBIs in Africa. However, several limitations identified in the previous review remain. For example, explicit reporting of a sustainability framework in the planning or measuring sustainability remains minimal, with only 50% of the studies published since 2015 reporting the use of a sustainability framework compared to 56% in the previous review led by Iwelunmor et al. [31]. Similarly, 52% of the interventions in this review, compared to 51.2% (very close proportions) in the 2015 review, provided a clear definition of sustainability. This may reflect the conceptual and methodological limitations that exist in framing and measuring sustainability, as documented in other reviews [9, 78]. Nonetheless, this updated review adds valuable insights to the existing literature on the sustainability of public health interventions in African countries and suggests progress, with the increasing attention and efforts devoted to understanding and documenting the long-term impact and effectiveness of interventions in this region.

### The primacy of key people and partnerships

One of the prominent facilitators identified in the synthesis is the active involvement of health systems, healthcare providers, and communities. Community engagement and partnership are important, consistent with other studies suggesting that it is critical to understand the link between the proposed intervention/program and the intended audience's strategic priorities, needs, and resources [31, 79, 80]. The value of person-centered and community-focused approaches to foster active community engagement was considered integral. The importance of centering the end-users and communities in intervention development and implementation is not new, but the challenge lies in ways to execute this that are long-lasting and beneficial to the communities. More broadly, engagement approaches that involve co-creation [81] and acknowledging the strengths and uniqueness of people within the context can help to maximize the fit of interventions and higher potential for sustainability [82, 83]. For instance, how participatory approaches such as human-centered design [84, 85] and crowdsourcing [86] fit into implementation science and how they can guide active community engagement can be explored to foster and continue active community engagement, an important facilitator of sustainability [87].

### Building/supporting capacity

This review also highlights the need to build capacity and train local implementers and community members to facilitate sustainability. This is consistent with findings from other reviews [13] and fields of work that herald capacity building and training as critical for sustainability [3, 88, 89]. This will involve reconfiguring EBIs implemented in African countries to include a training component to strengthen the in-house workforce. Capacity building, however, should also involve ‘capacity listening’ so that the community is engaged in ways that make the planning more iterative and more responsive to needs. Done in a culturally responsive manner, capacity-building ensures that communities and in-house implementers have the skills, resources, and confidence to continue implementing the intervention or program in the long-run [90]. Intervention sustainability may often be hinged on internal skills for intervention implementation or ensuring that equipment can be used. Therefore, capacity-building that leverages existing strengths and resources while building skills and resources that can last should be at the forefront of planning for programs to last.

### Integrating planning

**Fig. 5:**
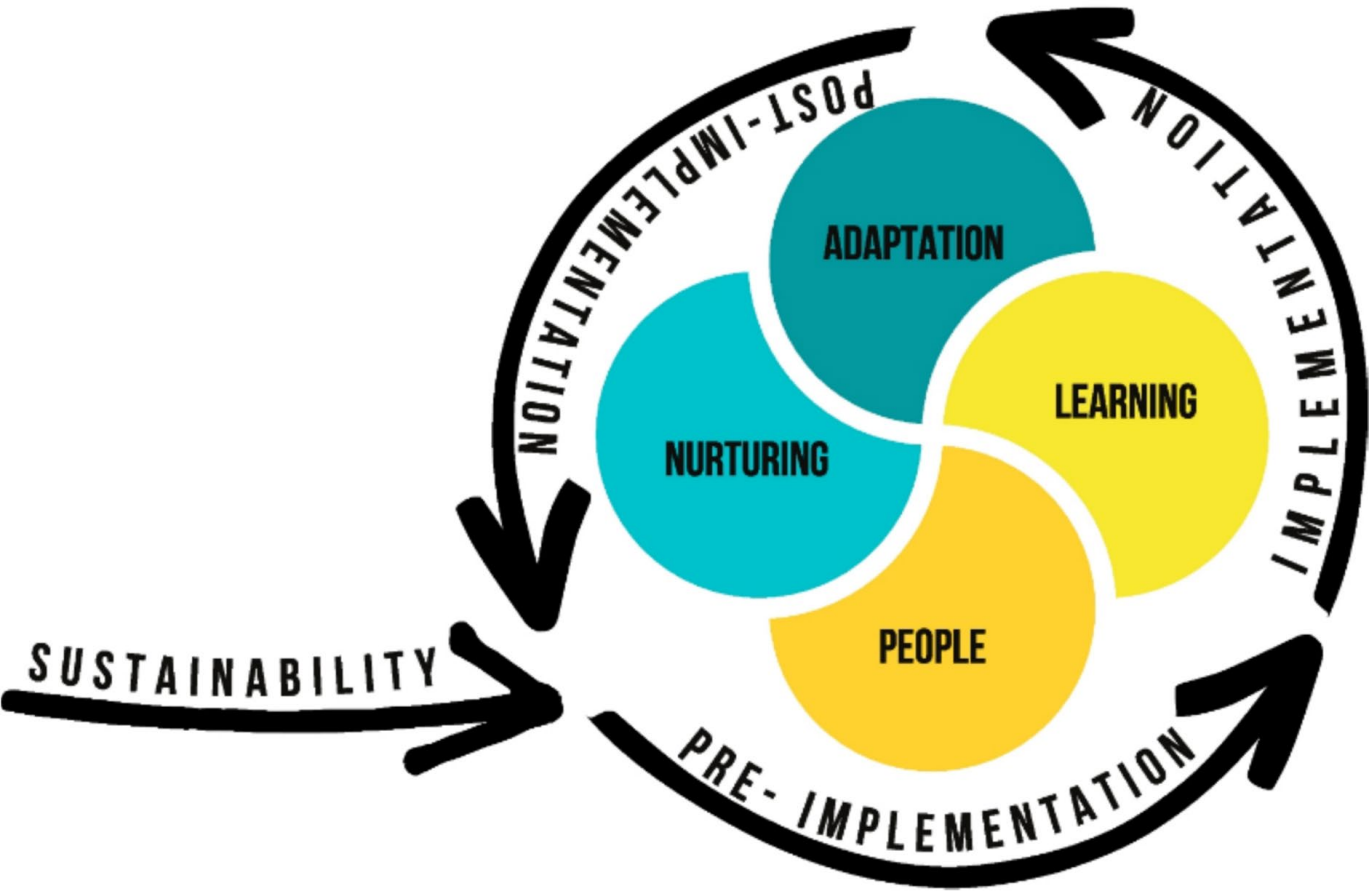
PLAN (People, Learning, Adaptation, and Nurturing) as determinants of sustainability, from Iwelunmor et al. [14]

Since the prior review, more attention has been focused on addressing the issue of why planning matters for sustainability. Even when addressed fully, one of the limitations we observed is that attention to sustainability remains focused on the end of the project rather than at the beginning and throughout the project's lifecycle. On an encouraging note, recent research focusing on how researchers conceptualize sustainability has started to note that the mechanisms guiding sustainability cannot simply be ascribed to the end of a project, as planning from the beginning and throughout the lifecycle of a project matters [91–93]. Decisions about sustainability are not static but dynamic and iterative and include how interventionists learn, adapt, and nurture the core values of their projects over time. The sustainability of interventions in the region can be improved by using a framework that guides how People Learn, Adapt, and Nurture (PLAN) the core values of an intervention by Iwelunmor and colleagues [14]. PLAN, developed in the context of over six years of ongoing research in Nigeria, argues for the need to plan and develop more practical and realistic strategies that foster sustainability and equity (See Fig. 5).

Our findings illustrate how the process of planning for sustainability throughout the lifecycle of an intervention should take into consideration the people that matter. That engagement begins with learning throughout the implementation process, the adaptations or changes made along the way, and the key elements they choose to nurture and sustain. PLAN's key components, alongside attention to the interactions between interventions/innovations, practice settings, intervention fit, and the broader ecological contexts in which implementation occurs, may move the field forward [79].

### The role of context and new areas for research

Intervention sustainability is influenced by various cultural, social, economic, and political factors, which vary by context. The activities involved in planning for sustainability are also diverse, shaped by both internal and external processes unique to each setting. This highlights the need to explore how sustainability can be fostered amidst these complex interplay of factors [94]. A systems approach could provide a holistic understanding of the interconnections within these complex systems, offering insights into how sustainability can be better planned and achieved [95]. Understanding the relationship between the potential determinants of sustainability and how it changes over time may require understanding the mechanisms of sustainability. As we map the mechanisms for implementation [96, 97], it may be critical to map the mechanisms for sustainability and how varying contexts impact it [98]. This would include identifying the core determinants, including the barriers and facilitators, the mediators and moderators of sustainability, which may comprise multiple factors in the context [98, 99]. A systems approach to sustainability may help us further distill sustainability levels, as one size does not fit all, and to account for the complex and dynamic systems where interventions are implemented and need to be sustained. Future studies can focus on developing a framework to identify *“if”* and *“how”* to sustain interventions.

In addition, the lack of consistency in measuring sustainability across the studies reviewed underscores the need for valid, reliable, and context-relevant measures that tap into crucial factors that influence sustainability in the region [100]. Although most of the studies used qualitative methods, none of the quantitative studies included validated measures for sustainability. While qualitative reports on sustainability are invaluable, validated scales in quantitative interventions are likely to offer an assessment of sustainability across contexts and common assessment factors that may need to be strengthened to plan for sustainability. Moreover, important sustainability tools such as the Program Sustainability Assessment Tool (PSAT) [101] and Clinical Sustainability Assessment Tool (CSAT) [102], which have high psychometric properties, exist but were conceived and validated outside of the African region. Therefore, there is a need for tools that account for the unique implementation context that is shared in African countries. This involves developing new contextually relevant tools or adapting existing tools to the African context.

### Implications

This review presents directions for maximizing public health outcomes by highlighting factors that influence the sustainability of interventions in African countries. For EBIs to be sustainable, it is critical to involve key communities of interest, such as policymakers, the government, and the end-users, to provide technical and financial support to implement and sustain these interventions. Hence, the funders of projects need to account for the time and resources required to build authentic partnerships and collaborations with the long-term goal of fostering sustainability [103]. Funders should also build in resources and recommendations for investing in capacity building and support for local staff and community members.

Although more studies examine intervention sustainability, consistent, region-specific metrics are lacking. Researchers should develop contextually relevant, standardized measures incorporating community engagement, capacity building, policy support, and equity considerations to address health disparities [104]. Future research should focus on creating application-oriented sustainability frameworks to strengthen planning for sustainability from the onset, which centers on the unique context, nurtures the assets within these contexts, and improves the reporting of sustainability planning and outcomes.

In addition, this review shows increasing numbers of studies reporting on “how” interventions are being sustained; however, consistent with the other literature, a consistent metric for sustainability specific to the region is still lacking. There were inconsistencies in terms of operationally defining and measuring sustainability. Researchers should strive to develop contextually relevant and standardized metrics for measuring sustainability outcomes. This will involve a holistic approach beyond simply measuring continued implementation but also considering factors such as community engagement, capacity building, policy support, and long-term health impact. Similarly, the current sustainability assessments do not consider equity as a praxis; an equity-focused lens will allow for deliberate considerations and planning for sustainability in resource-limited settings. Adopting an equity-focused lens in planning for sustainability is critical to addressing health disparities [104]. With this recommendation, the authors also acknowledge that one size does not fit all; in some situations, a predefined metric may not be appropriate but can provide a guiding frame for other measures and indicators that may work better. Alongside this, there is a need to crystallize how to evaluate sustainability. Future research can explore how sustainability is measured in the region and the use of an application-oriented sustainability framework to strengthen planning for sustainability from the onset, which centers the unique context and nurtures the assets within these contexts. Similarly, better reporting of activities involved in planning for and actual sustainability is recommended.

Further, limited funding is continually cited as a barrier to sustainability and now requires action beyond the typical forms of funding. Innovative strategies to generate continued funding for research beyond the lifecycle of grants should be considered. Strategies such as including economic strengthening as part of the intervention, utilizing crowdfunding strategies [105, 106], and integrating intervention within corporate social responsibility (CSRs) of private sectors [107] could be increasingly explored from the onset of program implementation to sustain implementation beyond the dedicated funded period.

### Study strengths and limitations

Our study has some strengths. First, this study contributes to the growing body of knowledge on sustainability that public health stakeholders in African countries can utilize. This includes program implementers, policymakers, funders, and researchers, providing them with valuable insights and strategies for effectively implementing evidence-based interventions. Second, our search strategy and review process were comprehensive and rigorous, following the PRISMA checklist with PROSPERO protocol registration [33]. For example, we conducted a thorough reference list search of all published articles, including relevant systematic reviews on sustainability, to ensure we captured any studies that might have been missed in the initial database search. In addition, to enhance the reliability of our findings, each included study underwent data abstraction review by more than one author.

The strengths of the review notwithstanding, the findings of this study should be interpreted considering some limitations. Despite our efforts to conduct a thorough search of the literature, like any systematic review, it is possible that some relevant articles were not captured in our review. We acknowledge the potential for missing pertinent information. However, the presence of a substantial number of duplicated studies obtained in the search provides a degree of confidence that the main papers indexed have been included in the review. We were further able to synthesize the findings from the included studies, offering an overview of the existing sustainability landscape. Furthermore, the review of the studies was limited to the information published in the literature. We did not include gray literature or reports there; it is possible that we missed findings from non-peer-reviewed publications, which could have offered more comprehensive documentation of additional aspects of sustainability.

## Conclusions

This review highlights progress in documenting the sustainability of public health interventions in Africa. Key factors for sustaining these interventions include meaningful community engagement, early stakeholder planning, and multisectoral collaboration. Financing remains a significant challenge, suggesting the need for innovative funding mechanisms, such as crowdfunding and leveraging private sector resources. The review also stresses the importance of people, learning, adaptation, and nurturers (PLAN) in promoting sustainability. While this review advocates for long-term sustainability, it acknowledges that some interventions, like those for pandemics (e.g., Ebola, COVID-19), may be time-bound, given the urgency of actions necessary for containment.

## Supplementary Information

Supplementary Material 1.

Supplementary Material 2.

Supplementary Material 3.

## Data Availability

The authors are willing to share the raw data tables that informed the summary tables included in this manuscript.

## Abbreviations

AE: Alexis Engelhart
AM: Ashley Murphy
BPT: Bryce P. Takenaka
CO: Chisom Obiezu-Umeh
CA: Collins Airhihenbuwa
DO: David Oladele
DS: Donna Shelley
EA: Ebenezer Adeoti
GO: Gbenga Ogedegbe
IDC: Innocent David Chinaemerem
IO: Ifeoma Obionu
JI: Juliet Iwelunmor
LAB: Lateef Akeem Blessing
OA: Onyekachukwu Anikamadu
OE: Oliver Ezechi
OF: Olufunto Olusanya
PM: Patrick Murphy
PP: Pranali Patel
SM: Stacey Mason
SN: Susan Nkengasong
TG: Titilola Gbajabiamila
TO: Temitope Ojo
TS: Thembekile Shato
UN: Ucheoma Nwaozuru
