# Supplementary material for: The sustainability of health interventions implemented in Africa: an updated systematic review on evidence and future research perspectives
Source: Implement Sci Commun. 2025 Apr 8;6:39. doi: 10.1186/s43058-025-00716-x (PMC11980204; doi:10.1186/s43058-025-00716-x)
Supplement: Supplementary file 3 — Supplementary Material 3. [file 43058_2025_716_MOESM3_ESM.docx]

Quality Assessment

Two authors (UN, CO) appraised the quality of all retained studies independently using Hawker’s Quality Assessment Checklist.[1] This quality appraisal tool allows for appraising all types of empirical studies, including quantitative, qualitative, and mixed methods.[1] Each selected article was appraised based on the nine domains of Hawker’s Quality Assessment Checklist:[1] 1) abstract/title; 2) introduction and aims; 3) method and data; 4) sampling; 5) data analysis; 6) ethics and bias; 7) results; 8) transferability and generalizability and 9) implications and usefulness. For each study, the nine domains were assigned a score between 1-4 [4=good; 3=fair; 2=poor; 1=very poor]. The score for each study was calculated by summing across the domains. An overall quality rating of low, medium, or high was assigned to each study based on the cut-off used by Braithwaite et al.[2] and Zipfel et al.[3] [*high quality*: 30–36 points; *medium quality*:24–29 points; and low quality: 9–23 points]. No study was excluded even after quality appraisal, irrespective of its methodological quality, to increase the comprehensiveness of the systematic review by allowing the consolidation of all available evidence.

References

1. Hawker S, Payne S, Kerr C, Hardey M, Powell J: **Appraising the evidence: reviewing disparate data systematically**. *Qualitative health research* 2002, **12**(9):1284-1299.

2. Braithwaite J, Ludlow K, Testa L, Herkes J, Augustsson H, Lamprell G, McPherson E, Zurynski Y: **Built to last? The sustainability of healthcare system improvements, programmes and interventions: a systematic integrative review**. *BMJ open* 2020, **10**(6).

3. Zipfel N, Horreh B, Hulshof CT, de Boer AG, van der Burg-Vermeulen SJ: **The relationship between the living lab approach and successful implementation of healthcare innovations: an integrative review**. *BMJ open* 2022, **12**(6).
